# Genes of the Unfolded Protein Response Pathway Harbor Risk Alleles for Primary Open Angle Glaucoma

**DOI:** 10.1371/journal.pone.0020649

**Published:** 2011-05-31

**Authors:** Mary Anna Carbone, Yuhong Chen, Guy A. Hughes, Robert N. Weinreb, Norman A. Zabriskie, Kang Zhang, Robert R. H. Anholt

**Affiliations:** 1 Department of Genetics, North Carolina State University, Raleigh, North Carolina, United States of America; 2 W. M. Keck Center for Behavioral Biology, North Carolina State University, Raleigh, North Carolina, United States of America; 3 Institute for Genomic Medicine, University of California San Diego, San Diego, California, United States of America; 4 Department of Ophthalmology and Vision Science, Eye and ENT Hospital, Shanghai Medical School, Fudan University, Shanghai, China; 5 Hamilton Glaucoma Center and Department of Ophthalmology, University of California San Diego, San Diego, California, United States of America; 6 Department of Ophthalmology and Visual Sciences, Moran Eye Center, University of Utah School of Medicine, Salt Lake City, Utah, United States of America; 7 Molecular Medicine Research Center and Department of Ophthalmology, West China Hospital, Sichuan University, Sichuan, China; 8 Department of Biology, North Carolina State University, Raleigh, North Carolina, United States of America; Ohio State University Medical Center, United States of America

## Abstract

The statistical power of genome-wide association (GWA) studies to detect risk alleles for human diseases is limited by the unfavorable ratio of SNPs to study subjects. This multiple testing problem can be surmounted with very large population sizes when common alleles of large effects give rise to disease status. However, GWA approaches fall short when many rare alleles may give rise to a common disease, or when the number of subjects that can be recruited is limited. Here, we demonstrate that this multiple testing problem can be overcome by a comparative genomics approach in which an initial genome-wide screen in a genetically amenable model organism is used to identify human orthologues that may harbor risk alleles for adult-onset primary open angle glaucoma (POAG). Glaucoma is a major cause of blindness, which affects over 60 million people worldwide. Several genes have been associated with juvenile onset glaucoma, but genetic factors that predispose to adult onset primary open angle glaucoma (POAG) remain largely unknown. Previous genome-wide analysis in a *Drosophila* ocular hypertension model identified transcripts with altered regulation and showed induction of the unfolded protein response (UPR) upon overexpression of transgenic human glaucoma-associated myocilin (MYOC). We selected 16 orthologous genes with 62 polymorphic markers and identified in two independent human populations two genes of the UPR that harbor POAG risk alleles, BIRC6 and PDIA5. Thus, effectiveness of the UPR in response to accumulation of misfolded or aggregated proteins may contribute to the pathogenesis of POAG and provide targets for early therapeutic intervention.

## Introduction

POAG is the most prevalent form of glaucoma, and a major cause of irreversible blindness [1]. It is a complex inherited disorder that affects over 2 million people in the United States and is estimated to afflict over 60 million people worldwide [2]. POAG is often, but not always, accompanied by ocular hypertension and characterized by progressive loss of retinal ganglion cells, atrophy of the optic nerve, and visual field loss [3], [4]. Over 20 genetic loci and mutations in four genes have been associated with some cases of POAG, including CYP1B1, MYOC (myocilin), OPTN (optineurin) and WDR36, but the molecular pathogenesis of the disease remains largely unknown [5], [6], [7]. To date, there have been two POAG genome-wide association (GWA) studies [8], [9]. The first has identified intergenic polymorphic markers associated with POAG in a Japanese population, but these markers could not be linked to annotated genes [8], while another GWA study has implicated a common variant near CAV1 and CAV2 in POAG [9]. It is not uncommon for different GWA studies to discover different marker associations for the same phenotype. Replication can be predicted only when the assumption that common alleles underlie a common disease is valid. However, when disease status represents a large mutational target, i.e. many rare alleles can predispose to the same disease phenotype, it is unlikely that different populations capture the same low frequency alleles, and populations of different genetic backgrounds may harbor population-specific variants.

To address the challenging multiple testing problem inherent in GWA studies and to identify potential candidate genes involved in POAG pathogenesis, we explored a comparative genomics strategy. We developed a transgenic *Drosophila* model to analyze whole-genome transcription profiles from heads of flies that express human MYOC in their eyes. Overexpression of MYOC results in distortion of the ommatidia, accompanied by periodic fluid discharge through the lenses [10], [11]. Western blotting identified the accumulation of myocilin aggregates and a genetically expressed fluorescent indicator revealed activation of the UPR [10], [11]. Although the anatomy and physiology of the fly eye is different from the human eye [12], we reasoned that stress pathways, such as the unfolded protein response (UPR) [13], would be evolutionarily conserved and that human orthologues of genes that undergo altered regulation in the *Drosophila* ocular hypertension model could be identified as candidate genes that might harbor risk alleles for POAG in human populations. Based on results from the previous study in the *Drosophila* MYOC-induced ocular hypertension model [11], we selected genes based on three criteria (Table S1): (1) they showed differential expression on *Drosophila* microarrays (FDR<0.005) [11]; (2) they have human orthologs; and (3) they have known functions in the UPR, ubiquitination, proteolysis, or oxidative-stress pathways [14], [15], [16]. We also included MYOC [17], OPTN [18], and NTE [10], because these genes had previously been implicated in glaucoma. We employed a case-control study using two different Caucasian populations (Salt Lake City, Utah and San Diego, California). We identified several SNPs significantly associated with POAG and two of these (rs2754511 and rs11720822) were identified in both of our study populations, implicating UPR in the pathogenesis of POAG.

## Results

### Statistical Analysis of SNP Associations with POAG

**Figure 1:**
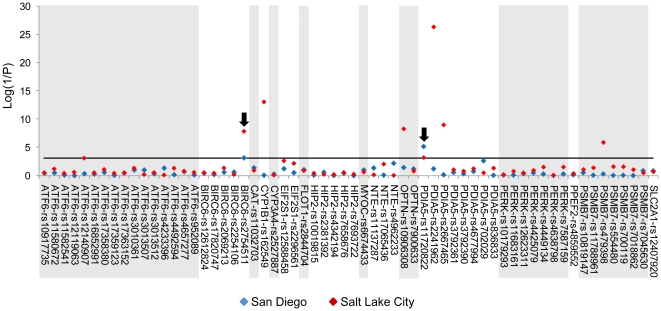
Association between POAG and candidate gene tagging rsSNPs. The graph shows the genotypic P-values (Log(1/P); y-axis) from likelihood ratio Chi-squared tests of associations with candidate gene SNPs (x-axis) from 2 populations (San Diego (blue) and Salt Lake City (red)). The black arrows indicate SNPs that were significant in both the San Diego and Salt Lake City cohorts. The black horizontal line indicates the Bonferroni adjusted P-value (P = 0.0008).

Mutations in MYOC have been associated with juvenile onset glaucoma, and with some cases of POAG [17], [19]. OPTN has also been associated with various forms of glaucoma, including POAG and normal tension glaucoma [20]. We did not detect associations between MYOC with POAG in either the Salt Lake City or San Diego population. This is perhaps not surprising as MYOC mutations have been associated with only ∼3–4% of POAG cases [7]. We did, however, find an association between OPTN and POAG in the Salt Lake City population (rs10906308, P = 5.84×10–9; Figure 1 and Table S2). The same SNP in the San Diego population, however, does not reach statistical significance (P = 0.04). Similarly, SNPs in CYP1B1 and PSMB7, which encodes proteasome subunit beta 7, are significantly associated with POAG in the Salt Lake City population only (Figure 1 and Table S2). Thus, previously reported associations with POAG of at least some candidate genes were replicated in at least one of our populations.

We identified SNPs associated with POAG in both the Salt Lake City and San Diego populations in PDIA5 (PDIR), a protein disulfide isomerase which facilitates the formation of disulfide bonds during protein folding and is associated with the UPR (Tables S3 and S4), and in BIRC6 (BRUCE, APOLLON), which encodes a ubiquitin ligase that protects against apoptosis (Tables S5 and S6) [21], [22]. In PDIA5, rs11720822 is significantly associated with POAG in both the Salt Lake City and San Diego populations (Tables S3 and S4). Additional SNPs in PDIA5 are significantly associated with POAG in only one of the populations; rs2241962 and rs2667465 are significant in the Salt Lake City population only (Tables S2 and S3).

### Haplotype Analysis

We next examined linkage disequilibrium (LD) among the 8 PDIA5 SNPs (rs11720822, rs2241962, rs2667465, rs3792361, rs3792390, rs4677994, rs702029, rs836833) and among the 5 BIRC6 SNPs (rs12612824, rs17820747, rs2069213, rs2254106, and rs2754511) that were genotyped in both cohorts. Patterns of LD are similar among the SNPs for both PDIA5 and BIRC6 in both populations and between cases and controls (Figures S1 and S2).

**Figure 2:**
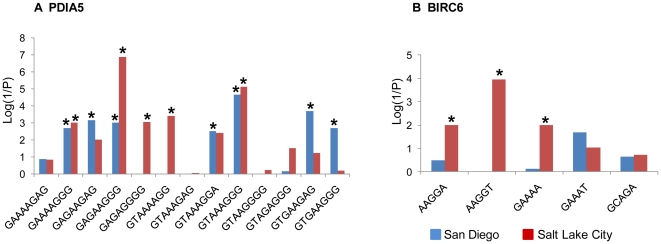
Association of PDIA5 (A) and BIRC6 (B) haplotypes with POAG. The graphs show the Fisher's P-values (Log(1/P); y-axis) with the haplotypes (x-axis) from 2 populations (San Diego (blue) and Salt Lake City (red). The PDIA5 haplotypes are composed of the following rsSNPs: rs11720822, rs2241962, rs2667465, rs3792361, rs3792390, rs4677994, rs702029, rs836833. The BIRC6 haplotypes are composed of the following rsSNPs: rs12612824, rs17820747, rs2069213, rs2254106, and rs2754511. Haplotypes with frequencies <0.03 were excluded from the analysis. Haplotypes significantly associated with POAG are indicated with an asterisk (Bonferroni adjusted P<0.004 for PDIA5; Bonferroni adjusted P<0.01 for BIRC6).

We next compared the distributions of the haplotypes composed of these PDIA5 and BIRC6 SNPs (Figure 2, Tables S7, S8, S9, S10). The frequency of the GTAAAGGG haplotype of PDIA5 was higher in POAG cases than controls in both the Salt Lake City and San Diego populations (odds ratio = 2.4 (95% CI 1.6–3.5) in the Salt Lake City population; odds ratio = 6.0 (95% CI 2.4–16.3) for the San Diego population; Tables S7 and S8), implicating it as a risk haplotype in both populations. In addition to SNPs in PDIA5, we identified one SNP in BIRC6, rs2754511, that was significantly associated with POAG in both the Salt Lake City (P = 1.6×10^−8^) and San Diego population (P = 7.5×10^−4^) (Figure 1 and Table S2). Haplotype analysis indicates that the GAAAT haplotype of BIRC6 appears to be protective in each population (Tables S9 and S10).

All POAG-associated SNPs are in introns, except rs12612824, which tags BIRC6 in an intergenic region. Sequence variants in non-coding gene regions may affect transcription efficiency, alternative splicing and/or mRNA stability. Large LD blocks in the human genome, however, make it difficult to identify not only causal SNPs, but, depending on the extent of linkage disequilibrium, also causal genes. However, identification of risk alleles in genes located on different chromosomes that contribute to a common cellular pathway instills confidence that PDIA5 and BIRC6 are likely to be indeed causally associated with POAG risk.

## Discussion

Previous GWA studies on glaucoma identified intergenic SNPs [8] and implicated a common variant near CAV1 and CAV2 in POAG [9] but failed to identify genes previously associated with glaucoma, such as MYOC and OPTN [6], [7]. Although these studies used large numbers of unaffected controls, the actual number of POAG cases was restricted to 418 and 1263, respectively. This limitation combined with the large number of SNPs surveyed may have limited the detection power of these studies. We have circumvented the large multiple testing problem inherent in GWA studies by using a comparative genomic approach that enabled us to perform a genome-wide transcriptional analysis in *Drosophila* [11] and to select, based on the results of these studies, 16 evolutionarily conserved candidate genes with human orthologs. Thus, whereas a previous GWA study surveyed 303,117 SNPs across the genome with about three times more POAG samples than our study [9], we interrogated about 5,000 times fewer polymorphic markers, thereby gaining substantial power for identifying alleles significantly associated with POAG.

We genotyped 62 SNPs in a Salt Lake City population and in a San Diego replicate population and identified two SNPs associated with POAG in both populations. We also observed SNPs in OPTN, CYP1B1 and PSMB7 associated with POAG, but only in the Salt Lake City population. This observation supports the general notion that different populations may harbor distinct risk alleles for the same disease.

The two SNPs significantly associated with POAG in both populations are in two different genes, BIRC6 and PDIA5, both of which function to reduce endoplasmic reticulum (ER) stress. BIRC6 is an ubiquitin-carrier protein involved in the regulation of apoptosis [22], while PDIA5 is an ER chaperone that is induced during ER stress [23]. The identification of two alleles which both function to alleviate ER stress, strongly implicates the UPR as a pathogenic mechanism for POAG in these populations. Studies in tissue culture [24] and mice [25] have also indicated that sequestration of myocilin in the ER may be associated with POAG. Furthermore, misfolded mutant myocilin forms intracellular aggregates that fail to be transported from the ER to the cytosol; as a result, components of the UPR, including BiP and PERK are induced, with subsequent apoptotic cell death [16]. One can speculate that apoptotic cell death in the trabecular meshwork would impair the ability of the anterior chamber to regulate intraocular pressure and lead to POAG resulting from ocular hypertension, whereas apoptotic cell death resulting from failure of the UPR in retinal ganglion cells themselves could conceivably be a contributing factor to normal tension glaucoma.

Components of the UPR have been associated with several common neurodegenerative diseases, including Alzheimer's disease and Parkinson's disease [26], [27]. Association of PDIA5 and BIRC6 (as well as PSMB7 and CYP1B1 in the Salt Lake City population) with POAG indicates that adult-onset glaucoma belongs to the same category of late onset neurodegenerative diseases. Previous studies in trabecular meshwork cell cultures and the *Drosophila* ocular hypertension model have also been suggestive of the UPR in the pathology of glaucoma [11], [14], [28]. Our results indicate that DNA variants that compromise the efficiency of the UPR may give rise to a common mechanism for the pathogenesis of POAG, and implicate the UPR as a future target for therapeutic intervention.

## Materials and Methods

### Study Population

This study was approved by the Institutional Review Boards of the University of California San Diego and the University of Utah. All participants provided written informed consent prior to participation in the study and are of Caucasian ethnicity. POAG diagnosis was made by the presence of specific signs of glaucomatous optic nerve damage on an eye examination plus visual field defects; intraocular pressure (IOP) was not considered in this definition. The normal controls were from the same geographic areas with a healthy optic nerve and cup to disc ratio <0.3. None of the control individuals had elevated IOP. The mean ages of our San Diego cohorts for controls and cases were 69 and 76, respectively. The mean ages of our Salt Lake City cohorts for controls and cases were 68 and 74, respectively. Males and females were evenly distributed among the sample sets. DNA was extracted from blood samples by using the FlexiGene DNA kit (Qiagen) for genotype analyses.

### SNP Selection and Genotyping

Based on results from a previous study in the *Drosophila* MYOC-induced ocular hypertension model [11], we selected 16 candidate genes, which were homologs of differentially expressed genes (FDR<0.005) in the *Drosophila* model and play a role in the UPR, ubiquitination, proteolysis, or oxidative-stress pathways (Table S1). Tagging SNPs were chosen from genotyped SNPs of Utah residents with ancestry from Northern and Western Europe (CEU) in the HapMap (www.hapmap.org) and NCBI dbSNP (http://www.ncbi.nlm.nih.gov/SNP/) databases (minor allele frequency ≥0.05) using Tagger software [29]. We genotyped 207 POAG cases and 270 normal controls in the population from Salt Lake City, Utah. For the replicate population from San Diego, California, we genotyped 471 POAG cases and 151 normal controls. To avoid spurious associations due to population admixture, we restricted our cohorts exclusively to subjects of Caucasian ethnicity. Genotyping was performed with an Illumina GoldenGate Oligo Pool All (OPA) assay, which is based on hybridization of allele-specific oligonucleotides (Table S1) to biotinylated DNA immobilized to streptavidin-coated paramagnetic particles. The allelic variant that matches the genomic sequence is preferentially extended to produce a synthetic template which is then amplified with fluorescently labeled universal primers and hybridized to VeraCode beads through an address sequence [30]–[32].

Genotype calling was performed using BeadStudio Version 3 (Illumina, Inc.). The accuracy of the genotype calls was manually evaluated using the software's clustering algorithm and quality scores. Following removal of genotypes with low quality scores, the genotyping error rate was estimated to be 1.4%.

### Statistical Analysis

To test the 62 SNPs for associations with disease status, we computed the likelihood ratio chi-square (χ^2^) for each SNP, by population, using JMP 8 (Table S2). We used a conservative Bonferroni corrected P-value of 0.0008 as the threshold. HWE tests, allele frequencies, genotype frequencies, LD (D' and r^2^), and haplotype analyses were performed using the online software SHEsis (http://analysis.bio-x.cn/myAnalysis.php) [33], [34].

## Supporting Information

Figure S1 **Linkage disequilibrium (D') among 8 rsSNPs in the PDIA5 gene.** Controls (A and C) and POAG (B and D) for both the Salt Lake City (A and B) and San Diego (C and D) populations are shown. Linkage disequilibrium analyses (D' and r^2^) were performed using the online software, SHEsis (http://analysis.bio-x.cn/myAnalysis.php) (31, 32). The D' values are highlighted as follows: red (1.0<D'<0.75); dark pink (0.74<D'<0.65); light pink (0.64<D'<0.10) and white (0.09<D'<0).
(TIF)

Figure S2 **Linkage disequilibrium (D') among 5 rsSNPs in the BIRC6 gene.** Controls (A and C) and POAG (B and D) for both the Salt Lake City (A and B) and San Diego (C and D) populations are shown. Linkage disequilibrium analyses (D' and r^2^) were performed using the online software, SHEsis (http://analysis.bio-x.cn/myAnalysis.php) (31, 32). The D' values are highlighted as follows: red (1.0<D'<0.75); dark pink (0.74<D'<0.65); light pink (0.64<D'<0.10) and white (0.09<D'<0).
(TIF)

Table S1 Sequences of rsSNPs genotyped in the Salt Lake City and San Diego cohorts.
(DOC)

Table S2 Associations of SNPs with POAG in the Salt Lake City and San Diego cohorts.
(DOC)

Table S3 χ2 tests for frequency distributions of alleles and genotypes in PDIA5 (Salt Lake City, Utah).
(DOC)

Table S4 χ2 tests for frequency distributions of alleles and genotypes in PDIA5 (San Diego, California).
(DOC)

Table S5 χ2 tests for frequency distributions of alleles and genotypes in BIRC6 (Salt Lake City, Utah).
(DOC)

Table S6 χ2 tests for frequency distributions of alleles and genotypes in BIRC6 (San Diego, California).
(DOC)

Table S7 Estimated PDIA5 haplotype frequencies and association significance for the Salt Lake City population.
(DOC)

Table S8 Estimated PDIA5 haplotype frequencies and association significance for the San Diego population.
(DOC)

Table S9 Estimated BIRC6 haplotype frequencies and association significance for the Salt Lake City population.
(DOC)

Table S10 Estimated BIRC6 haplotype frequencies and association significance for the San Diego population.
(DOC)
