# Supplementary figures and images for: Genes of the Unfolded Protein Response Pathway Harbor Risk Alleles for Primary Open Angle Glaucoma
Source: PLoS One. 2011 May 31;6(5):e20649. doi: 10.1371/journal.pone.0020649 (PMC3105107; doi:10.1371/journal.pone.0020649)

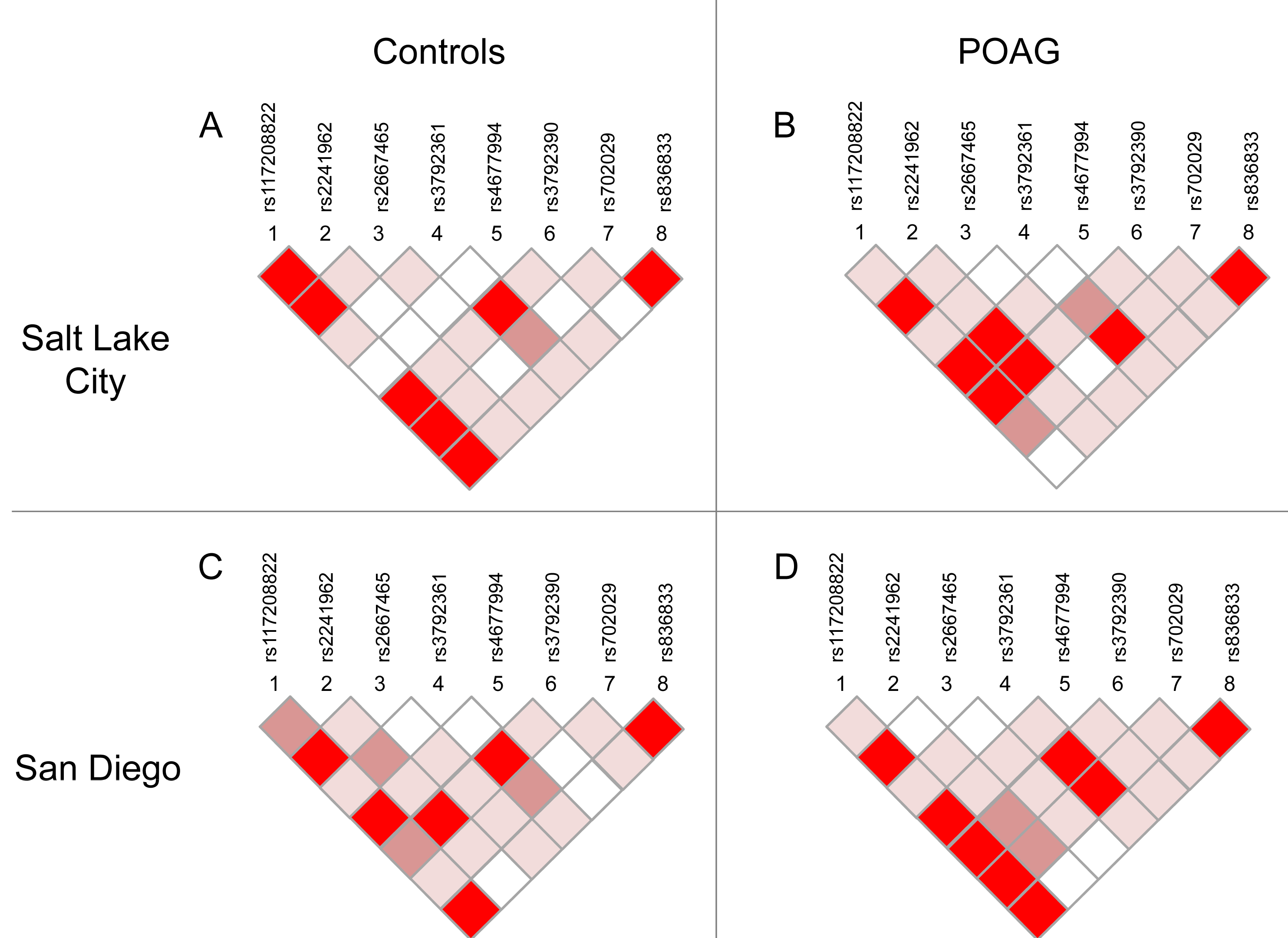

Supplement: Figure S1 — Linkage disequilibrium (D') among 8 rsSNPs in the PDIA5 gene. Controls (A and C) and POAG (B and D) for both the Salt Lake City (A and B) and San Diego (C and D) populations are shown. Linkage disequilibrium analyses (D' and r2) were performed using the online software, SHEsis (http://analysis.bio-x.cn/myAnalysis.php) (31, 32). The D' values are highlighted as follows: red (1.0<D'<0.75); dark pink (0.74<D'<0.65); light pink (0.64<D'<0.10) and white (0.09<D'<0). (TIF) [file pone.0020649.s001.tif]

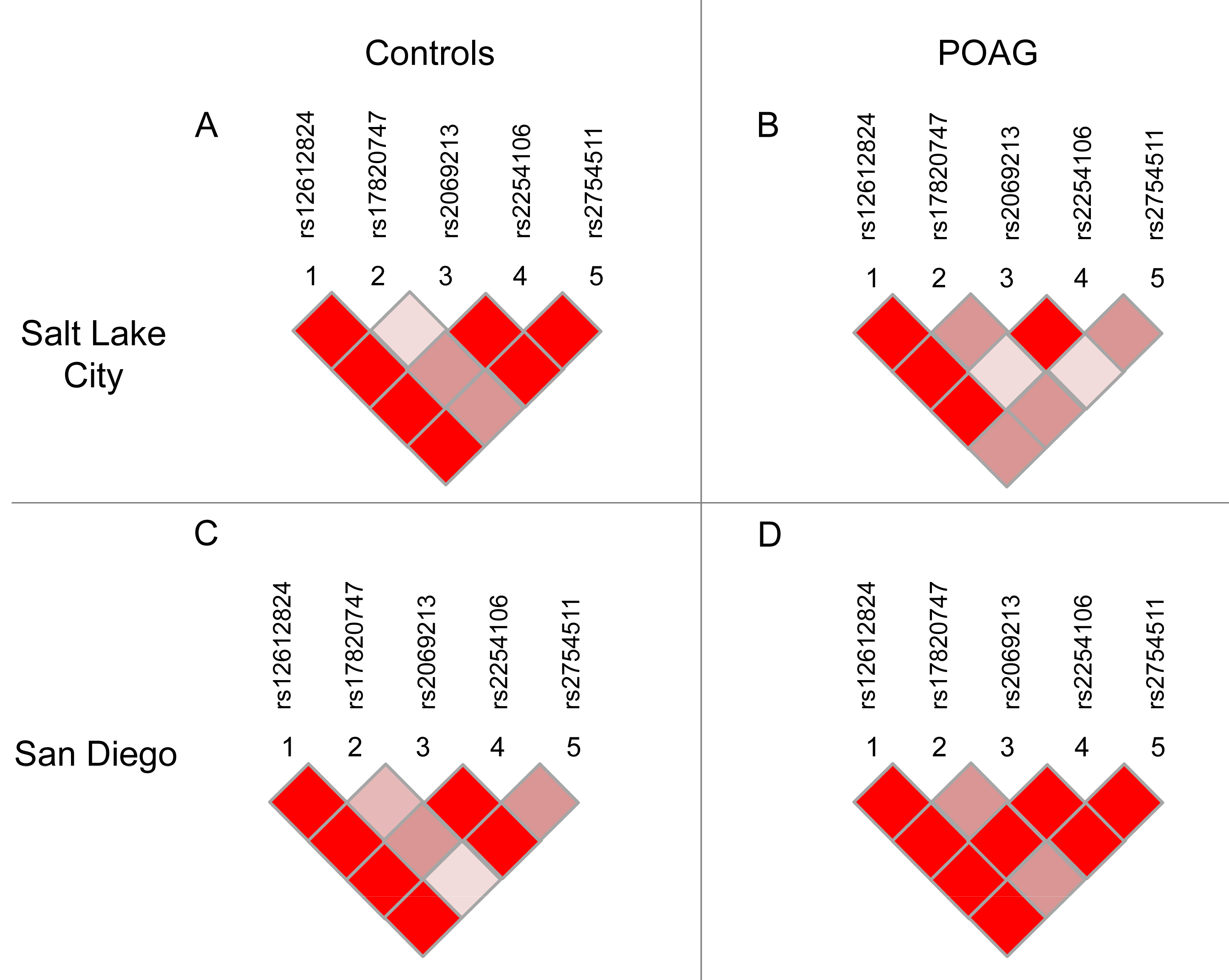

Supplement: Figure S2 — Linkage disequilibrium (D') among 5 rsSNPs in the BIRC6 gene. Controls (A and C) and POAG (B and D) for both the Salt Lake City (A and B) and San Diego (C and D) populations are shown. Linkage disequilibrium analyses (D' and r2) were performed using the online software, SHEsis (http://analysis.bio-x.cn/myAnalysis.php) (31, 32). The D' values are highlighted as follows: red (1.0<D'<0.75); dark pink (0.74<D'<0.65); light pink (0.64<D'<0.10) and white (0.09<D'<0). (TIF) [file pone.0020649.s002.tif]
