# Supplementary material for: Genes of the Unfolded Protein Response Pathway Harbor Risk Alleles for Primary Open Angle Glaucoma
Source: PLoS One. 2011 May 31;6(5):e20649. doi: 10.1371/journal.pone.0020649 (PMC3105107; doi:10.1371/journal.pone.0020649)
Supplement: Table S1 — Sequences of rsSNPs genotyped in the Salt Lake City and San Diego cohorts. (DOC) [file pone.0020649.s003.doc]

**TABLE S1: Sequences of rsSNPs genotyped in the Salt Lake City and San Diego cohorts**

| **rsSNP** | **Tagged Gene** | **SNP** | **Sequence** | **Chromosome** | **Coordinate** |
| --- | --- | --- | --- | --- | --- |
| rs17358380 | ATF6 | [T/C] | ATAAGTAGTAAGTAAACAATAACTCATGTGGCAATTAGTCTTTAGTGAGCTCCCAAAATG[T/C]GCCAGGTATACAGTGGTACTTATAACTTCCAAGCATAATGAGAGCATTTGTAGTCTTTTG | 1 | 161538634 |
| rs12140907 | ATF6 | [A/G] | TCAGACCCCGCCACCCCCTTCACTAGTAAAGCAGTTTCTGAAGTTCTAGGGCTTAAAATG[A/G]AAGCTGATAAGAAAGAAAGAATGTTTAGAGGCCTTTGAATTCCTAAGCATATAGATATAA | 1 | 161714068 |
| rs11582541 | ATF6 | [T/C] | gaggtggaacagtttcatcccaaaaccaatccccccacatatccaacgcccttgacatcc[A/G]actcctgctcccccaacccatcccatggaaaaactgtcttccaaaaatgagtccctggtg | 1 | 161540374 |
| rs952089 | ATF6 | [T/C] | GAGGAAGTACATTTCATTTTCTGAGACAGACAAATAAGACAGGTGGATATATGTGGCATT[A/G]ACAGAAGTGGAGAGGGAGGAAGTTATATATTGTATGATGGCTTTAATTTTCTCAATTAAC | 1 | 161555290 |
| rs11580672 | ATF6 | [A/G] | CAACCACTGTATCTTTTCACACAGAGCTTCAGAAACTATTGGTCCTCTTTCAAGTAGTCA[T/C]TACTTTGGTGGCTTGCAGCCCTACACACTACACTAGTAAGCTTTCTGCCTGCAGAACATC | 1 | 161541939 |
| rs17359123 | ATF6 | [T/G] | GTTGTTCATGTCTCCATTAGCATAGCAGTTAGAGTGTTTTGCTGCATTTATCTCCTTTGA[T/G]AAAGTTAGGTGCTTATGAGGTTGTGAACTCAATTCTCAGGAGTATAGGTTAACTTCTGGT | 1 | 161610967 |
| rs4492594 | ATF6 | [T/C] | GAGCCTAGTTGAGGGTCAGGGCCCACCTGAGCATCCCTATTCTTTCTCCACTCTGTTCCA[A/G]GGGCCTAGCAGTTCTCACTTTTCATGCTGCACACACAAGGGATGAAAGCATGTGTCCTCA | 1 | 161647368 |
| rs10917735 | ATF6 | [A/G] | TCACTGCTAACTCGTCTTTCCTGTTTGAGCATAGGTGTAAGGATTGCAGGACATTAGAAG[A/G]GGGATTTTCAAACCTCAGTATTTATCAGAATCACCTGGAGGACTAAAATGGATTATTGCC | 1 | 161597791 |
| rs3013512 | ATF6 | [C/G] | CAATTGTTTGTAAAATTACTTAATACTTTCCTCCCTTGTAATAAAGTAATTAAATCTCAC[C/G]ACCCAGCATTTCTGTTTTGATCTCCCAGAGAAAAGCCTCGAAAAATTGTTGCATAATTGA | 1 | 161580543 |
| rs17363152 | ATF6 | [T/C] | TCCTTTTTTTTTTTTTCCTGGTTAACAGGAAATTTCTAAGAAATGATAGACATCTGACAC[A/G]CGGTCTCAATCCAGAAATTCAGATACAACAGAAGTGAAGTCTACACATTAATTGATACTC | 1 | 161601182 |
| rs12119063 | ATF6 | [A/C] | tgctgtcatgtctctgattctctcttctgattttctattccactttaaaaaatcctcatg[T/G]ttacattaggcccactcacataattcgggataatctctttattttcagatcaactgatta | 1 | 161720197 |
| rs3013507 | ATF6 | [A/G] | AATCAGTGTAACCTCTACTATGTAAAAATGTATATGCGTTAGTAAGCTATTGCTGCATAA[T/C]GAACTAGTCCTCCCCCAAAACTTAATGATTCATCAAAAAACCATTTATTTCACGCATAAT | 1 | 161612588 |
| rs4657277 | ATF6 | [T/G] | AAACTTGATAATTTCTTTTCTATTCCCTAGAATGCTGGGCCTGGCTAAGATCTGGTGTTG[T/G]CATTACATGTTGAGGAATAATCAATTGTCAGTTAAATTTACTGTGAATAAATGAGATATG | 1 | 161683599 |
| rs3010361 | ATF6 | [T/C] | ATGCCTGATTCTTTCTTCTCCACTTCATGGACATCCACTCATCCTCCTGTTATGACTCCA[A/G]TGTGATCTTCTCTTGCTCAGCCCTGACTGACTTCCCTGGACAGAGCTGGCCTCCCGCTCC | 1 | 161644160 |
| rs16852991 | ATF6 | [T/C] | GTACTTAGCTATATGTAGAGAAAATATTTAATTTCAAGTTCAACTGACATATTTCAAGGA[A/G]CTAATCCATTTTATGACAAAGCAAGGGAAATTGAAATATGCACCTTGCAGGCTGAAGTTA | 1 | 161595522 |
| rs4233396 | ATF6 | [T/C] | GTACAGCAATCCCTCTGACTTCACAAAATAAAACCTGTTCTCCCAAGGCTAGAGTGGATC[T/C]ATGGGCTAGGGGTACAAATAGGGATGTATACCTCACACATACAAATAAAAGTAGGACCCC | 1 | 161685373 |
| rs2754511 | BIRC6 | [A/T] | GGTGTAACCCTAAACAGAAAAGGAAAAAAAAATGGCTTTCTGTAATTTCACACTATTTTT[A/T]GTGCATATACAATGAATTTTTATTAAAAAGGATAGTTATGATATCTCTCTCTCTAATAAT | 2 | 32623661 |
| rs17820747 | BIRC6 | [A/C] | AGTTCCAGTAAATTGAAGATAAAGGATAGTGGAATGTATAGTTTTGCTAATGTGGCATTG[A/C]TAGAGCAGTTTATTTCCATTTTACAATACAAAATTATACAAGATGATTGCAAATATTATT | 2 | 32555191 |
| rs2254106 | BIRC6 | [T/C] | CTCACTTCCAGTATTAGTTATACATAATAAATCCTAAAGAATCTTCATTTTCATCTCTGT[A/G]TTCATTCAAAAATTATTTTCCTTGTGCTGACAAAACTGTTGGGCATCACTGGGGGGATGT | 2 | 32622301 |
| rs12612824 | BIRC6 | [T/C] | AGGCTCCCTACCCTCCACTGGGGGCTCTGAAGGGACTGAGAGGACCCTCCAAATCAATCA[T/C]TTGAGGCTGAAAAGAGAGAAAAAAATGAGACTGTTTTGAAACAGGGCAAATAAAAAAAAA | 2 | 32433263 |
| rs2069213 | BIRC6 | [A/G] | CGTTATGCTAGGTATTCATTATGTGCAAACCGTTTAGTACTAATTTTTTCTAACCCTGGA[T/C]ACGTTTCTTACACTTTTATCTCCTTTGGTATTCATATATTTTAAGATAAAATCATTTCTT | 2 | 32596588 |
| rs11032703 | CAT | [A/G] | TCTCCTTTATTCAATATCTTTTTTCAAACAGCTCACAGATCTTTTGTGGTTTTGTAAGTG[T/C]CTACTCCCCACCGTATTGCCTTCTAGAGAAGAGGCAGGAATTTATTTTAATGTACAGGAC | 11 | 34426232 |
| rs162549 | CYP1B1 | [T/A] | GGCAAAAGGAAAGCTGTGTTTATATGGAAGAAAGTAAGGTGCTTGGAGTTTACCTGGCTT[A/T]TTTAATATGCTTATAACCTAGTTAAAGAAAGGAAAAGAAAACAAAAAACGAATGAAAATA | 2 | 38148960 |
| rs2527887 | CYP3A4 | [T/A] | TTAATCCACAGCTCTCCTGTCAGCCCACAGTTCTTGAATTTTGGCCTCTTTTATGTCCAC[A/T]TGAATGTAAATATACTTCAGAATTTCCACCCAGAATCTTTGGAATCTAATTTCTTCAATC | 7 | 99388890 |
| rs2296561 | EIF2S1 | [A/T] | AGCAGATTAATAAAATCTCAAGCATCCTAATAGTTGTGGGTTCTGTAGGTACATTCATAA[A/T]TGAATTTTTGTCTTTCTCTTTTAGATTAATCTAATAGCTCCTCCTCGGTATGTAATGACT | 14 | 66918892 |
| rs12588458 | EIF2S1 | [A/G] | TCTCAAGATTAGGTATACCTATTCAATAAAAGAAAGGAATATTTGGGAGTATTGGGGATA[A/G]GAGAAGCTAGACATTGTGAATTCAAGGAAAAAGAATTATGAGAGGAGGATGCATTTTAGT | 14 | 66900609 |
| rs2844704 | FLOT1 | [T/G] | TGGCATACAGCTTTGCAACTCAAACTGTGTTCTACCCAGCAGCAGCTGCAGCATCACTGC[T/G]AAGCTTGTTAGAAATACAAAATTGCAGGCCCTGGCCCTGACCAGCTGAATCAGAACCTGC | 6 | 30813992 |
| rs7693722 | HIP2 | [G/C] | AATCCTGAGCAGGACTCATTCCCCATCTGCCAGATATGACCAATCATGCTAGTTATGTTT[C/G]TTTCACCCTTACAAAGACAACTTTAGAGCAGTACAGAAATTAGATAACTATCAAATAACT | 4 | 39302484 |
| rs7658676 | HIP2 | [T/C] | ATTATACTGTATCAGTAACTACATCTGTGAGTTTCCTAAGCACACAGGGATTTAAATTCT[T/C]GTAGTTATAACTAACGGCAAAAAATTATACCCTCAGATAAATTGCTTTTAAAATACAGGC | 4 | 39279527 |
| rs2381392 | HIP2 | [T/G] | CAAGTAATAGTGGCCATGAAGTGGGATGGATGAGTGGGGAGCAATAGAAAGGGACATGTT[T/G]ACATTTTGCTCCATATTTCTACATTATTTCAGGCTTTCATGGTCTAGATATTCACATATT | 4 | 39263240 |
| rs4342194 | HIP2 | [T/C] | AAAAAGATCTGGGAAGATACACACCAATTCATCTAGGAAAGTAGGAAATAAAAGCTAAGT[A/G]TGGGGAAAGGAGTTCAGCCTTACTTTTAATGTATTGATATTTTTTGAAAACACATATTCA | 4 | 39266759 |
| rs10019815 | HIP2 | [T/C] | TAGACAGGGATGTATGTGCGTATACAGGCATCAGTTTTTCCTGGTTCCTTGACATAGCCA[T/C]GTCTTATTTAATTTGAAATAACAAGTGCCTAGCACAGTGCCTGACCCATGATATGAGGTT | 4 | 39236085 |
| rs6674433 | MYOC | [T/G] | GTAGCTGTGGAGCCTGGGTCTGGTGTACAGGCATGTGTAGGGTTACCACTGCTCCAGGGA[T/G]CAGGTTGCTGACATGACTGCTGCAGTGGTAGCTCCAGTATTAGAGTGGCCACAGACCCTG | 1 | 171408011 |
| rs11137287 | NTE | [T/C] | GCTTCGGCCCGAACGCGGCTCTCGCGGTCTGATGTCTCGTCCCGAGCTTCGGCCCGAACG[T/C]GGCTCTCTGATGTGGATTTATTTTTATTTTTCCTGCAGCTTTTCCTTGGGAATCCCTTTC | 9 | 139502526 |
| rs17065436 | NTE | [A/G] | CATTTGAGGCGGAATGGGGGAGGCCATTGCTAGTTCCATATGGCCTTAGCCTTGGGAAAA[A/G]GCACTGGTGAGGTTCTGGCTCTGGGCCAGAAGGGAGGTGGAGCAGACACAGGCCACCTGT | 9 | 139502683 |
| rs4962233 | NTE | [A/G] | TCGTGGCCTCCTGGATTTCATGACACCCACACACGCTTGGACATAGTCCATGTTCTATGA[A/G]CATCTCTCCGTGTCATTATATTCAAGTCCACACCGTCCCTTTAAAAGGGCCCCATTAGCT | 9 | 139512156 |
| rs10906308 | OPTN | [A/G] | TTGCATTTCCCTGATCACTAATGGGAAAGAGTACTTTTTCAAGTGTTTTTGGCCTTTGAG[A/G]TATCCTCTTTTGTGAAGTGCCTTATCAAGCCTGCCTTTTTTTTTTTTTTTTTTTTTTTTT | 10 | 13210473 |
| rs7900633 | OPTN | [A/G] | CACTTCTGCATTATACCTGTTTTCTAAGTGAATTTGGGTGTGTGACACATAGATACAAAA[A/G]GTTCAAAAATGAATCCTATGGTTTATCAGTGTTTTCTGCTTCGTAAGATTGCCATCACCC | 10 | 13188473 |
| rs2667465 | PDIA5 | [A/G] | GTCTCTGCTTGTGAGGGCTGCTCCACTAATACCTGCTCCCCTTTCCTGCCTCGGCTGAAC[T/C]TCCGTCTAGGCCTGCTTGGCCTGTTTATCATCTAGGACTCTATGCTCAGCTTATGCCTGG | 3 | 124357488 |
| rs836833 | PDIA5 | [A/G] | TCTGGGGATCCTGAGGACCCCCTGCTCTTCCTCTCCCCTGTGCTACAGGGCTTCTTGGGG[A/G]CCACGTGGCTCTACCCTTGCCTACCCCCTGACTCATCGACCCATTTTCCTTAGCAACAGG | 3 | 124336959 |
| rs3792390 | PDIA5 | [A/G] | GGCTTTTCTTTGTCTTCCCTCCCCTGATTCCTACTCCAGTCCCACTGTCCATCTTGACCA[T/C]GGGCCCTGCCAGCAGCTGGCCTAACAGCACCATCCATATCATGGAGGGTATGGGGAGAGT | 3 | 124342771 |
| rs702029 | PDIA5 | [T/C] | CAGTCAGAAGGGCCAGAGAGGGCTTCTGAAGGCGACATCATACCCGGCTGCGGAGGGAAG[T/C]GGGTGGCAGAGTTCCATTGTGTCAGCCACTTCACTCCCTCTGGATTTTCCAGCCCTGGCT | 3 | 124330967 |
| rs4677994 | PDIA5 | [T/C] | CAGCTGTGAAATGGGACAGCGATATGCGTTCTGCCTTCTTTCCAGGTCTGTGATAAGGAT[T/C]GGGTAAATGAAAGTTCTTTATAAAATAGGAAATACAATTTAAATGTGAGACACCATTCTC | 3 | 124296743 |
| rs3792361 | PDIA5 | [T/C] | TGTCCTATCAGGTGCTTGCTACTTTCTTTTCCTCGGGGCTTAAGGTCATTGCTTAACCAC[A/G]TCTGCTTTCTTTCCTGCTCTCCTCCCCATCTGCTTGATTTCTCTGGAGCAGTCACAGCTT | 3 | 124298562 |
| rs2241962 | PDIA5 | [A/T] | TTTAGTTAGATTAATTTTATTTCAGAATGGTTTTCTTCCTGTGTTTACAATTATTTCTTT[A/T]AAAAAAAAATCCTGAACTCCGTTCAAAAGCTGAATCTTATTAAGATGCTGCAGGAAACCA | 3 | 124304168 |
| rs11720822 | PDIA5 | [A/G] | GCATGTTCCCCCGAGTGGTTGGGACATTGAGGGTGGATTTCCTTGCCCATCTAAAAATCT[T/C]TCCTTATGGCTGCCTCCCCACTCCCCTGGCTTCTTGCAGCCCTGAGGCCCCCCCGCCCCC | 3 | 124351731 |
| rs4449134 | PERK | [T/C] | CCCTTCTAGAATAGTCCCTTCCTCTTAGGAATTGCTTGGGAGATGTTAGTAGAAAGAACC[A/G]TGGCCCAGGTGCATTTTATCTTGTTAGTGCCAGCCTATCCCAGAAGTAGTCAGGGTAGGG | 2 | 88628076 |
| rs7587159 | PERK | [G/C] | gtcaaagttgaaaggtcaggcagatggagggagctttgaggtgggacaaaaaccacaggg[C/G]tctggtgttctggagatcacgtgaagaaaggtgacagggaggagggagcaacctgcggat | 2 | 88624526 |
| rs4425079 | PERK | [A/G] | gctgagttcaagtgcaaaatcatctgactctaaagCTTTTTCCTCTCTGCATTTAGCCTC[T/C]CCCATGTAAGTCCAGCACTGAAGGTCTTCATTGCACAGCCCCAGATGCCTTTCCAGCCTG | 2 | 88597157 |
| rs12623311 | PERK | [A/G] | GGTCTAGAGTGTCCCGTGGAATAAAATAAACCTCCAATTGTTTAAGCcaatgtttctcaa[A/G]cttacctgtacattagaatcacctgtggagcttaaaaagtatatatatatatactgattc | 2 | 88626255 |
| rs11683161 | PERK | [T/C] | CTGTTACACATAGGTGGTTAAATGGTTTTAACCAATGATAGTTACACTCAAGATTTTGCT[A/G]GGAAGAGTAGGGAAAAACAAAATTTTATCTCTAAATACTACATATTACAAATGAATACAA | 2 | 88589123 |
| rs4638798 | PERK | [A/G] | AAGGTCTTCATTGCACAGCCCCAGATGCCTTTCCAGCCTGACTTTTAAATATCCCCTATA[T/C]AGTCAAAGAGCATTATGGAGGGTCTAGCAGGCTCAGAGGAAGTCAAGAGGAAAAAGGCAG | 2 | 88597238 |
| rs10179293 | PERK | [G/C] | TTGGAAGATCTATTATGGTTGGAGCTCCAGGAAATCATGAAGAAAAAAATGACAAGGCAC[C/G]CTGCCCTCTGGAAATTTATGGGGAAAACAGGTCTCAGCTACCGATGATCGCTCCACCACC | 2 | 88582526 |
| rs4859552 | PPEF2 | [A/G] | gtttgcactctgatattttcttagagtatgcctttagaaatggaattttctaaaattgct[A/G]aataactaaaaatggtcataacagtttatactctcatcagcagtgtataagtgtaataat | 4 | 76897600 |
| rs7018862 | PSMB7 | [A/G] | ATAAGGACCGACATTAATTCAAGAATTATTTGTTGAGTGCCTGCTAAGTCCTAGAGTGGT[A/G]AATAAAAGAGACAGGGCTCCTCAGGTGCCTGCCCCTGTGAAGCTTACAATCTAAAGAAAG | 9 | 128121830 |
| rs7045630 | PSMB7 | [A/G] | CTAAAAAAAAGAGACATTTCAGCTAAAGGTATAAGAAAATGATGCAAATAAAATCAGAAC[T/C]GAAAAACTGAGAGTTCCTGACCTCTTCACTGGATTAGTCAAATGCCTCTTCCTTTCACTT | 9 | 128175299 |
| rs554480 | PSMB7 | [T/C] | CCAATGAGCAAATAAATAAATGAATGAGTAATGGTCAGAAAAAGTTTCACTAAGGGGCTA[T/C]GTAACCTGGATTTTAAGGATGAGAAAAGTTTTACTTAAAAGTTGGGGGACTGGGTATAGG | 9 | 128147964 |
| rs700119 | PSMB7 | [A/T] | tcaaaaaaaaacaaaacaaaaCACCAGGGTCACATCTTCCATTCCCCTCAAACTTTCTCC[A/T]ATTTCCCAGTCAGACAGGTGCCACCACTCAGAATCCTGACATCATGCAAGCACGTAGTCT | 9 | 128135146 |
| rs11788961 | PSMB7 | [A/G] | TGCTCTTATTGAGTTTAAGTGGCTCACAGTTGCCCGCTGTCTGGCCTGGGCCAAGTGTGT[A/G]TGTTTCCACTGCTGATGATCTAACATACCCAGAGGCCTATGAACTTCATTCTTGTTCATC | 9 | 128175508 |
| rs10819147 | PSMB7 | [A/G] | CTGTAGCCTCAGTGCTTAGCCCAAGTGGCTCCGTAGTCATCTGTGGACTCACTGTTCACG[T/C]GCCAGGCCCTCTTAGAGACCGGGAATGTAGGAGGATGCTTCAGGGGGAAGATGGCAAGGT | 9 | 128136295 |
| rs479398 | PSMB7 | [G/C] | AAGAGTCTCCCTCCCGCCTCCACCCGACCTGGCTGTCTCTGCTCCTGTCCCAGCAGAGAA[C/G]CCCAGCATCTACCCATGGCCAGCAAGACATCCTGTGGCACTCTCATCCTTCCCCTTCCTC | 9 | 128148547 |
| rs12407920 | SLC2A1 | [T/C] | CAGCATGGGGCAGGCAGGGGCAAGGATGGGGTCAACAGATGATGGGCATTTGCACTAGGA[T/C]ACTGCATTCACAAGACCAACTACTGAAGGGATATGTGTCTCAGGCTGTTCATTCTGGAAT | 1 | 43190368 |
