# Supplementary material for: Genes of the Unfolded Protein Response Pathway Harbor Risk Alleles for Primary Open Angle Glaucoma
Source: PLoS One. 2011 May 31;6(5):e20649. doi: 10.1371/journal.pone.0020649 (PMC3105107; doi:10.1371/journal.pone.0020649)
Supplement: Table S2 — Associations of SNPs with POAG in the Salt Lake City and San Diego cohorts. (DOC) [file pone.0020649.s004.doc]

**TABLE S2: Associations of SNPs with POAG in the Salt Lake City (Utah) and San Diego (California) cohorts**

| **rsSNP** | **Tagged Gene** | **χ2 (LR)** | **P-value** | **HWE χ2 (Controls)** | **HWE P-value (Controls)** | **HWE χ2 (POAG)** | **HWE P-value (POAG)** | **POPULATION** |
| --- | --- | --- | --- | --- | --- | --- | --- | --- |
| rs10019815 | HIP2 | 0.46 | 7.96E-01 | 1.46 | 2.26E-01 | 0.53 | 4.66E-01 | SD |
| rs10019815 | HIP2 | 1.80 | 4.07E-01 | 16.28 | 5.50E-05 | 0.20 | 6.60E-01 | SLC |
| rs10179293 | PERK | 0.53 | 7.69E-01 | 1.09 | 2.96E-01 | 0.53 | 4.65E-01 | SD |
| rs10179293 | PERK | 0.63 | 7.29E-01 | 0.58 | 4.40E-01 | 2.92 | 9.00E-02 | SLC |
| rs10819147 | PSMB7 | 2.44 | 2.96E-01 | 1.41 | 2.35E-01 | 0.09 | 7.60E-01 | SD |
| rs10819147 | PSMB7 | 4.79 | 9.13E-02 | 1.46 | 2.30E-01 | 0.02 | 9.00E-01 | SLC |
| rs10906308 | OPTN | 6.56 | 3.76E-02 | 0.05 | 8.23E-01 | 2.32 | 1.27E-01 | SD |
| rs10906308 | OPTN | 37.92 | 5.84E-09 | 2.03 | 1.50E-01 | 0.18 | 6.70E-01 | SLC |
| rs10917735 | ATF6 | 1.95 | 3.78E-01 | 1.03 | 3.11E-01 | 1.31 | 2.52E-01 | SD |
| rs10917735 | ATF6 | 2.25 | 3.25E-01 | 0.25 | 6.20E-01 | 1.41 | 2.30E-01 | SLC |
| rs11032703 | CAT | 6.38 | 4.11E-02 | 0.20 | 6.57E-01 | 0.58 | 4.46E-01 | SD |
| rs11032703 | CAT | 4.07 | 1.30E-01 | 0.69 | 4.10E-01 | 1.21 | 4.00E-02 | SLC |
| rs11137287 | NTE | 6.23 | 4.44E-02 | 0.53 | 4.65E-01 | 0.05 | 8.21E-01 | SD |
| rs11137287 | NTE | 0.45 | 8.00E-01 | 2.42 | 1.20E-01 | 0.90 | 3.40E-01 | SLC |
| rs11580672 | ATF6 | 2.31 | 3.15E-01 | 1.59 | 2.08E-01 | 0.93 | 3.35E-01 | SD |
| rs11580672 | ATF6 | 5.28 | 7.14E-02 | 0.01 | 9.30E-01 | 3.71 | 5.00E-02 | SLC |
| rs11582541 | ATF6 | 0.55 | 7.59E-01 | 0.01 | 9.18E-01 | 1.91 | 1.67E-01 | SD |
| rs11582541 | ATF6 | 1.73 | 4.20E-01 | 0.19 | 6.60E-01 | 1.59 | 2.10E-01 | SLC |
| rs11683161 | PERK | 0.55 | 7.61E-01 | 0.06 | 8.11E-01 | 0.26 | 6.10E-01 | SD |
| rs11683161 | PERK | 3.37 | 1.86E-01 | 0.13 | 7.10E-01 | 5.32 | 2.00E-02 | SLC |
| rs11720822 | PDIA5 | 23.63 | 7.41E-06 | 4.50 | 3.40E-02 | 470.00 | 0.00E+00 | SD |
| rs11720822 | PDIA5 | 14.51 | 7.06E-04 | 0.12 | 7.30E-01 | 0.00 | 9.90E-01 | SLC |
| rs11788961 | PSMB7 | 0.35 | 8.39E-01 | 0.46 | 4.96E-01 | 3.14 | 8.00E-02 | SD |
| rs11788961 | PSMB7 | 6.20 | 4.50E-02 | 0.79 | 3.70E-01 | 2.44 | 1.20E-01 | SLC |
| rs12119063 | ATF6 | 0.10 | 9.53E-01 | 0.00 | 9.88E-01 | 0.02 | 8.93E-01 | SD |
| rs12119063 | ATF6 | 2.64 | 2.67E-01 | 0.96 | 3.30E-01 | 1.54 | 2.10E-01 | SLC |
| rs12140907 | ATF6 | 1.59 | 4.51E-01 | 0.32 | 5.71E-01 | 4.07 | 4.30E-02 | SD |
| rs12140907 | ATF6 | 14.08 | 8.76E-04 | 1.16 | 2.90E-01 | 7.97 | 4.80E-03 | SLC |
| rs12407920 | SLC2A1 | 3.92 | 1.41E-01 | 0.84 | 3.58E-01 | 0.13 | 7.23E-01 | SD |
| rs12407920 | SLC2A1 | 2.99 | 2.25E-01 | 0.11 | 7.40E-01 | 0.42 | 5.20E-01 | SLC |
| rs12588458 | EIF2S1 | 5.62 | 6.02E-02 | 5.98 | 1.45E-02 | 2.06 | 1.51E-01 | SD |
| rs12588458 | EIF2S1 | 11.88 | 2.63E-03 | 6.46 | 1.00E-02 | 0.93 | 3.30E-01 | SLC |
| rs12612824 | BIRC6 | 1.93 | 3.81E-01 | 0.21 | 6.48E-01 | 3.65 | 5.60E-02 | SD |
| rs12612824 | BIRC6 | 2.31 | 3.15E-01 | 0.58 | 4.50E-01 | 0.52 | 4.70E-01 | SLC |
| rs12623311 | PERK | 1.62 | 4.46E-01 | 1.45 | 2.29E-01 | 0.33 | 5.68E-01 | SD |
| rs12623311 | PERK | 2.06 | 3.58E-01 | 0.13 | 7.20E-01 | 2.08 | 1.50E-01 | SLC |
| rs162549 | CYP1B1 | 0.31 | 8.58E-01 | 0.07 | 7.90E-01 | 0.59 | 4.42E-01 | SD |
| rs162549 | CYP1B1 | 59.89 | 9.91E-14 | 1.32 | 2.50E-01 | 0.00 | 9.70E-01 | SLC |
| rs16852991 | ATF6 | 1.29 | 5.25E-01 | 1.61 | 2.05E-01 | 0.88 | 3.48E-01 | SD |
| rs16852991 | ATF6 | 2.25 | 3.25E-01 | 0.41 | 5.20E-01 | 1.13 | 2.90E-01 | SLC |
| rs17065436 | NTE | 0.46 | 7.93E-01 | 22.70 | 2.00E-06 | 70.91 | 0.00E+00 | SD |
| rs17065436 | NTE | 9.26 | 9.77E-03 | 25.32 | 0.00E+00 | 1.53 | 2.20E-01 | SLC |
| rs17358380 | ATF6 | 2.72 | 2.57E-01 | 1.79 | 1.81E-01 | 0.96 | 3.28E-01 | SD |
| rs17358380 | ATF6 | 4.83 | 8.93E-02 | 0.01 | 9.40E-01 | 3.38 | 7.00E-02 | SLC |
| rs17359123 | ATF6 | 0.33 | 8.47E-01 | 0.09 | 7.65E-01 | 2.06 | 1.51E-01 | SD |
| rs17359123 | ATF6 | 1.81 | 4.05E-01 | 2.35 | 1.30E-01 | 0.02 | 8.90E-01 | SLC |
| rs17363152 | ATF6 | 2.19 | 3.34E-01 | 1.03 | 3.11E-01 | 2.03 | 1.54E-01 | SD |
| rs17363152 | ATF6 | 2.17 | 3.39E-01 | 0.14 | 7.10E-01 | 1.66 | 2.00E-01 | SLC |
| rs17820747 | BIRC6 | 0.92 | 6.30E-01 | 0.88 | 3.49E-01 | 0.10 | 7.48E-01 | SD |
| rs17820747 | BIRC6 | 1.83 | 4.01E-01 | 1.52 | 2.20E-01 | 0.38 | 5.40E-01 | SLC |
| rs2069213 | BIRC6 | 2.76 | 2.52E-01 | 0.48 | 4.90E-01 | 1.90 | 1.68E-01 | SD |
| rs2069213 | BIRC6 | 6.03 | 4.92E-02 | 2.25 | 1.30E-01 | 2.05 | 1.50E-01 | SLC |
| rs2241962 | PDIA5 | 3.49 | 6.17E-02 | 62.01 | 0.00E+00 | 270.36 | 0.00E+00 | SD |
| rs2241962 | PDIA5 | 120.99 | 5.35E-27 | 1.26 | 2.60E-01 | 2.11 | 1.50E-01 | SLC |
| rs2254106 | BIRC6 | 2.93 | 2.31E-01 | 0.23 | 6.30E-01 | 3.09 | 8.00E-02 | SD |
| rs2254106 | BIRC6 | 0.77 | 6.82E-01 | 0.22 | 6.40E-01 | 1.06 | 3.00E-01 | SLC |
| rs2296561 | EIF2S1 | 2.39 | 3.02E-01 | 4.66 | 3.10E-02 | 2.46 | 1.17E-01 | SD |
| rs2296561 | EIF2S1 | 9.77 | 7.57E-03 | 2.27 | 1.30E-01 | 2.37 | 1.20E-01 | SLC |
| rs2381392 | HIP2 | 2.81 | 2.45E-01 | 4.95 | 2.60E-02 | 0.39 | 5.34E-01 | SD |
| rs2381392 | HIP2 | 0.87 | 6.47E-01 | 0.62 | 4.30E-01 | 0.46 | 5.00E-01 | SLC |
| rs2527887 | CYP3A4 | 1.27 | 5.31E-01 | 1.06 | 3.04E-01 | 0.20 | 6.52E-01 | SD |
| rs2527887 | CYP3A4 | 0.25 | 8.83E-01 | 0.26 | 6.10E-01 | 0.70 | 4.00E-01 | SLC |
| rs2667465 | PDIA5 | 0.69 | 7.07E-01 | 0.06 | 8.00E-01 | 0.38 | 5.38E-01 | SD |
| rs2667465 | PDIA5 | 41.14 | 1.17E-09 | 4.38 | 4.00E-02 | 12.78 | 3.50E-04 | SLC |
| rs2754511 | BIRC6 | 14.40 | 7.47E-04 | 1.08 | 3.00E-01 | 3.40 | 6.50E-02 | SD |
| rs2754511 | BIRC6 | 35.88 | 1.62E-08 | 3.25 | 7.00E-02 | 50.64 | 0.00E+00 | SLC |
| rs2844704 | FLOT1 | 4.83 | 8.93E-02 | 7.23 | 7.00E-03 | 0.01 | 9.24E-01 | SD |
| rs2844704 | FLOT1 | 3.88 | 1.44E-01 | 0.58 | 4.50E-01 | 3.25 | 7.00E-02 | SLC |
| rs3010361 | ATF6 | 4.58 | 1.01E-01 | 1.70 | 1.92E-01 | 0.56 | 4.53E-01 | SD |
| rs3010361 | ATF6 | 5.86 | 5.34E-02 | 1.07 | 3.00E-01 | 1.58 | 2.10E-01 | SLC |
| rs3013507 | ATF6 | 4.59 | 1.01E-01 | 4.03 | 4.47E-02 | 0.01 | 9.36E-01 | SD |
| rs3013507 | ATF6 | 0.75 | 6.88E-01 | 0.00 | 9.70E-01 | 0.73 | 3.90E-01 | SLC |
| rs3013512 | ATF6 | 0.51 | 4.76E-01 | 0.09 | 7.71E-01 | 0.48 | 4.90E-01 | SD |
| rs3013512 | ATF6 | 2.29 | 3.19E-01 | 1.10 | 2.90E-01 | 0.65 | 4.20E-01 | SLC |
| rs3792361 | PDIA5 | 2.67 | 2.63E-01 | 1.13 | 2.88E-01 | 0.12 | 7.34E-01 | SD |
| rs3792361 | PDIA5 | 4.59 | 1.01E-01 | 0.87 | 3.50E-01 | 0.01 | 9.10E-01 | SLC |
| rs3792390 | PDIA5 | 1.55 | 4.60E-01 | 0.60 | 4.40E-01 | 0.78 | 3.76E-01 | SD |
| rs3792390 | PDIA5 | 3.46 | 1.78E-01 | 0.78 | 3.70E-01 | 2.95 | 9.00E-02 | SLC |
| rs4233396 | ATF6 | 6.16 | 4.60E-02 | 3.76 | 5.30E-02 | 1.49 | 2.22E-01 | SD |
| rs4233396 | ATF6 | 0.34 | 8.44E-01 | 0.38 | 5.40E-01 | 0.32 | 5.70E-01 | SLC |
| rs4342194 | HIP2 | 0.15 | 9.28E-01 | 0.16 | 6.85E-01 | 2.23 | 1.35E-01 | SD |
| rs4342194 | HIP2 | 0.77 | 6.79E-01 | 0.70 | 4.00E-01 | 0.16 | 7.00E-01 | SLC |
| rs4425079 | PERK | 2.27 | 3.21E-01 | 0.76 | 3.85E-01 | 0.37 | 5.43E-01 | SD |
| rs4425079 | PERK | 4.08 | 1.30E-01 | 0.84 | 3.60E-01 | 2.93 | 9.00E-02 | SLC |
| rs4449134 | PERK | 1.35 | 5.09E-01 | 0.16 | 6.90E-01 | 0.19 | 6.63E-01 | SD |
| rs4449134 | PERK | 6.76 | 3.41E-02 | 3.10 | 8.00E-02 | 3.55 | 6.00E-02 | SLC |
| rs4492594 | ATF6 | 0.16 | 6.91E-01 | 0.18 | 6.74E-01 | 0.41 | 5.22E-01 | SD |
| rs4492594 | ATF6 | 3.75 | 5.28E-02 | 0.40 | 5.30E-01 | 0.06 | 8.00E-01 | SLC |
| rs4638798 | PERK | 0.15 | 9.26E-01 | 0.12 | 7.26E-01 | 0.85 | 3.57E-01 | SD |
| rs4638798 | PERK | 0.20 | 9.05E-01 | 0.05 | 8.30E-01 | 0.24 | 6.30E-01 | SLC |
| rs4657277 | ATF6 | 3.18 | 2.04E-01 | 0.30 | 5.82E-01 | 0.74 | 3.90E-01 | SD |
| rs4657277 | ATF6 | 3.51 | 1.73E-01 | 0.20 | 6.60E-01 | 1.68 | 1.90E-01 | SLC |
| rs4677994 | PDIA5 | 3.47 | 1.77E-01 | 0.94 | 3.32E-01 | 0.99 | 3.20E-01 | SD |
| rs4677994 | PDIA5 | 5.51 | 6.37E-02 | 0.41 | 5.20E-01 | 1.36 | 2.40E-01 | SLC |
| rs479398 | PSMB7 | 1.41 | 4.94E-01 | 35.50 | 0.00E+00 | 161.00 | 0.00E+00 | SD |
| rs479398 | PSMB7 | 26.88 | 1.46E-06 | 38.18 | 0.00E+00 | 2.57 | 1.00E-01 | SLC |
| rs4859552 | PPEF2 | 0.69 | 7.07E-01 | 0.10 | 7.57E-01 | 0.86 | 3.53E-01 | SD |
| rs4859552 | PPEF2 | 1.50 | 4.73E-01 | 0.68 | 4.10E-01 | 0.49 | 4.80E-01 | SLC |
| rs4962233 | NTE | 10.04 | 6.60E-03 | 0.13 | 7.24E-01 | 0.27 | 6.05E-01 | SD |
| rs4962233 | NTE | 0.03 | 9.87E-01 | 0.03 | 8.60E-01 | 0.00 | 9.50E-01 | SLC |
| rs554480 | PSMB7 | 0.22 | 8.94E-01 | 0.60 | 4.40E-01 | 2.55 | 1.10E-01 | SD |
| rs554480 | PSMB7 | 7.12 | 2.84E-02 | 0.66 | 4.10E-01 | 3.29 | 7.00E-02 | SLC |
| rs6674433 | MYOC | 4.59 | 1.01E-01 | 18.06 | 2.10E-05 | 1.41 | 2.34E-01 | SD |
| rs6674433 | MYOC | 2.80 | 2.47E-01 | 0.02 | 8.90E-01 | 1.66 | 2.00E-01 | SLC |
| rs700119 | PSMB7 | 0.32 | 8.53E-01 | 1.39 | 2.38E-01 | 8.50 | 3.55E-03 | SD |
| rs700119 | PSMB7 | 7.03 | 2.97E-02 | 1.06 | 3.00E-01 | 3.29 | 7.00E-02 | SLC |
| rs7018862 | PSMB7 | 0.08 | 9.62E-01 | 0.08 | 7.75E-01 | 0.08 | 7.81E-01 | SD |
| rs7018862 | PSMB7 | 4.68 | 9.62E-02 | 0.09 | 7.60E-01 | 2.37 | 1.20E-01 | SLC |
| rs702029 | PDIA5 | 12.06 | 2.41E-03 | 7.24 | 7.13E-03 | 5.46 | 1.90E-02 | SD |
| rs702029 | PDIA5 | 2.09 | 3.52E-01 | 0.24 | 6.30E-01 | 1.01 | 3.10E-01 | SLC |
| rs7045630 | PSMB7 | 3.97 | 1.38E-01 | 0.09 | 7.65E-01 | 0.00 | 9.79E-01 | SD |
| rs7045630 | PSMB7 | 2.11 | 3.48E-01 | 1.49 | 2.20E-01 | 2.18 | 1.40E-01 | SLC |
| rs7587159 | PERK | 1.35 | 5.10E-01 | 0.06 | 8.06E-01 | 0.44 | 5.09E-01 | SD |
| rs7587159 | PERK | 6.81 | 3.33E-02 | 2.06 | 1.50E-01 | 4.28 | 4.00E-02 | SLC |
| rs7658676 | HIP2 | 1.95 | 3.78E-01 | 3.58 | 5.80E-02 | 0.24 | 6.27E-01 | SD |
| rs7658676 | HIP2 | 2.39 | 3.02E-01 | 2.09 | 1.50E-01 | 0.21 | 6.50E-01 | SLC |
| rs7693722 | HIP2 | 0.00 | 9.98E-01 | 0.53 | 4.68E-01 | 1.30 | 2.54E-01 | SD |
| rs7693722 | HIP2 | 0.95 | 6.21E-01 | 0.13 | 7.10E-01 | 0.02 | 8.80E-01 | SLC |
| rs7900633 | OPTN | 5.48 | 6.47E-02 | 2.47 | 1.16E-01 | 1.19 | 2.75E-01 | SD |
| rs7900633 | OPTN | 3.52 | 1.72E-01 | 0.01 | 9.20E-01 | 1.40 | 2.40E-01 | SLC |
| rs836833 | PDIA5 | 0.32 | 8.53E-01 | 4.23 | 4.00E-02 | 7.56 | 6.00E-03 | SD |
| rs836833 | PDIA5 | 5.93 | 5.15E-02 | 7.12 | 7.60E-03 | 0.41 | 5.20E-01 | SLC |
| rs952089 | ATF6 | 0.63 | 7.28E-01 | 0.01 | 9.18E-01 | 2.08 | 1.50E-01 | SD |
| rs952089 | ATF6 | 2.52 | 2.84E-01 | 0.19 | 6.60E-01 | 2.88 | 9.00E-02 | SLC |
