# Supplementary material for: Genes of the Unfolded Protein Response Pathway Harbor Risk Alleles for Primary Open Angle Glaucoma
Source: PLoS One. 2011 May 31;6(5):e20649. doi: 10.1371/journal.pone.0020649 (PMC3105107; doi:10.1371/journal.pone.0020649)
Supplement: Table S3 — χ2 tests for frequency distributions of alleles and genotypes in PDIA5 (Salt Lake City, Utah). (DOC) [file pone.0020649.s005.doc]

**TABLE S3: χ2 tests for frequency distributions of alleles and genotypes in PDIA5 (Salt Lake City, Utah)**

| **rsSNP and allele definitions** | **Samples** | **Allele 1**  **(freq)** | **Allele 2**  **(freq)** | **Fisher’s P-value (χ2)** | **OR (95% CI)** | **Genotype 11**  **(freq)** | **Genotype 12**  **(freq)** | **Genotype 22**  **(freq)** | **Fisher’s P-value (χ2)** | **HWE P-value Controls (χ2)** |
| --- | --- | --- | --- | --- | --- | --- | --- | --- | --- | --- |
| rs11720822 | POAG | 385(0.93) | 29(0.07) | 1.7E-04 | 3.59 | 179(0.86) | 27(0.13) | 1(0.005) | 0.0009 | 0.73 |
| 1=G 2=A | Control | 525(0.98) | 11(0.02) | (14.2) | (1.8-7.3) | 257(0.96) | 11(0.04) | 0(0.000) | (14.1) | (0.12) |
| rs2241962 | POAG | 376(0.91) | 38(0.09) | 0 | 0.16 | 169(0.82) | 38(0.18) | 0(0.000) | 3.2E-15 | 0.26 |
| 1=T 2=A | Control | 328(0.61) | 208(0.40) | (107) | (0.1-0.2) | 96(0.35) | 136(0.50) | 36(0.130) | (105.2) | (1.25) |
| rs2667465 | POAG | 286(0.79) | 76(0.21) | 6.1E-05 | 1.89 | 105(0.58) | 76(0.42) | 0(0.000) | 8.0E-07 | 0.04 |
| 1=A 2=G | Control | 341(0.671 | 171(0.33) | (16.1) | (1.4-2.6) | 121(0.47) | 99(0.40) | 36(0.141) | (28.2) | (4.28) |
| rs3792361 | POAG | 320(0.78) | 92(0.22) | 0.04 | 0.71 | 124(0.60) | 72(0.35) | 10(0.049) | 0.10 | 0.35 |
| 1=A 2=G | Control | 448(0.83) | 92(0.17) | (4.2) | (0.5-1.0) | 188(0.69) | 72(0.27) | 10(0.037) | (4.6) | (0.87) |
| rs3792390 | POAG | 328(0.79) | 86(0.21) | 0.79 | 1.04 | 134(0.64) | 60(0.30 | 13(0.063) | 0.18 | 0.37 |
| 1=A 2=G | Control | 424(0.78) | 116(0.22) | (0.07) | (0.8-1.4) | 164(0.61) | 96(0.36) | 10(0.037) | (3.46) | (0.78) |
| rs4677994 | POAG | 381(0.93) | 31(0.07) | 0.11 | 0.69 | 175(0.85) | 31(0.15) | 0(0.000) | 0.13 | 0.52 |
| 1=G 2=A | Control | 483(0.89) | 57(0.11) | (2.60) | (0.4-1.0) | 217(0.80) | 49(0.18) | 4(0.015) | (4.02) | (0.40) |
| rs702029 | POAG | 331(0.80) | 83(0.20) | 0.36 | 0.86 | 130(0.63) | 71(0.34) | 6(0.029) | 0.37 | 0.63 |
| 1=G 2=A | Control | 417(0.78) | 121(0.22) | (0.83) | (0.6-1.2) | 163(0.63) | 91(0.34) | 15(0.062) | (2.00) | (0.24) |
| rs836833 | POAG | 280(0.75) | 92(0.25) | 0.03 | 1.43 | 107(0.57) | 66(0.36) | 13(0.070) | 0.05 | 0.008 |
| 1=G 2=A | Control | 434(0.81) | 100(0.19) | (4.74) | (1.0-1.9) | 183(0.67) | 68(0.26) | 16(0.060) | (5.96) | (7.12) |
