# Supplementary material for: Genes of the Unfolded Protein Response Pathway Harbor Risk Alleles for Primary Open Angle Glaucoma
Source: PLoS One. 2011 May 31;6(5):e20649. doi: 10.1371/journal.pone.0020649 (PMC3105107; doi:10.1371/journal.pone.0020649)
Supplement: Table S4 — χ2 tests for frequency distributions of alleles and genotypes in PDIA5 (San Diego, California). (DOC) [file pone.0020649.s006.doc]

**TABLE S4: χ2 tests for frequency distributions of alleles and genotypes in PDIA5 (San Diego, California)**

| **rsSNP and allele definitions** | **Samples** | **Allele 1**  **(freq)** | **Allele 2**  **(freq)** | **Fisher’s P-value (χ2)** | **OR (95% CI)** | **Genotype 11**  **(freq)** | **Genotype 12**  **(freq)** | **Genotype 22**  **(freq)** | **Fisher’s P-value (χ2)** | **HWE P-value Controls (χ2)** |
| --- | --- | --- | --- | --- | --- | --- | --- | --- | --- | --- |
| rs11720822 | POAG | 938 (0.99) | 2 (0.002) | 0.000001 | 0.062 | 469 (0.99) | 0 (0.000) | 1 (0.002) | 2.3E-06 | 0.03 |
| 1=G 2=A | Control | 292 (0.97) | 10 (0.03) | (22.93) | (0 - 0.3) | 142 (0.94) | 8 (0.05) | 1 (0.007) | (26.0) | (4.5) |
| rs2241962 | POAG | 536 (0.57) | 406 (0.43) | 0.32 | 0.87 | 65 (0.14) | 406 (0.86) | 0 | 0.06 | 0.00 |
| 1=A 2=T | Control | 171 (0.60) | 113 (0.40) | (0.98) | (0.7-1.1) | 29 (0.20) | 113 (0.80) | 0 | (3.68) | (62.0) |
| rs2667465 | POAG | 484 (0.51) | 458 (0.49) | 0.41 | 0.90 | 121 (0.26) | 242 (0.51) | 108 (0.23) | 0.71 | 0.80 |
| 1=G 2=A | Control | 147 (0.49) | 155 (0.51) | (0.67) | (0.7-1.1) | 35 (0.23) | 77 (0.51) | 39 (0.26) | (0.70) | (0.06) |
| rs3792361 | POAG | 757 (0.80) | 185 (0.19) | 0.15 | 1.26 | 303 (0.64) | 151 (0.32) | 17 (0.04) | 0.26 | 0.29 |
| 1=A 2=G | Control | 231 (0.76) | 71 (0.24) | (2.10) | (0.9-1.7) | 86 (0.57) | 59 (0.40) | 6 (0.04) | (2.7) | (1.13) |
| rs3792390 | POAG | 744 (0.79) | 198 (0.21) | 0.58 | 1.10 | 297 (0.63) | 150 (0.32) | 24 (0.05) | 0.45 | 0.44 |
| 1=A 2=G | Control | 234 (0.77) | 69 (0.23) | (0.31) | (0.8-1.5) | 89 (0.59) | 56 (0.38) | 6 (0.04) | (1.6) | (0.60) |
| rs4677994 | POAG | 845 (0.90) | 97 (0.10) | 0.13 | 1.45 | 377 (0.8) | 91 (0.19) | 3 (0.006) | 0.26 | 0.33 |
| 1=G 2=A | Control | 278 (0.93) | 22 (0.07) | (2.31) | (0.9-2.4) | 128 (0.85) | 22 (0.15) | 0 (0.000) | (2.7) | (0.93) |
| rs702029 | POAG | 725 (0.77) | 217 (0.23) | 0.98 | 1.00 | 270 (0.57) | 185 (0.39) | 16 (0.03) | 0.002 | 0.007 |
| 1=G 2=A | Control | 232 (0.77) | 70 (0.23) | (0.003) | (0.7-1.4) | 95 (0.63) | 42 (0.28) | 14 (0.09) | (12.9) | (7.24) |
| rs836833 | POAG | 748 (0.80) | 186 (0.20) | 0.63 | 0.93 | 309 (0.660 | 130 (0.28) | 28 (0.06) | 0.85 | 0.04 |
| 1=G 2=A | Control | 238 (0.79) | 64 (0.21) | (0.23) | (0.7-1.3) | 98 (0.65) | 42 (0.28) | 11 (0.07) | (0.33) | (4.22) |
