# Supplementary material for: Genes of the Unfolded Protein Response Pathway Harbor Risk Alleles for Primary Open Angle Glaucoma
Source: PLoS One. 2011 May 31;6(5):e20649. doi: 10.1371/journal.pone.0020649 (PMC3105107; doi:10.1371/journal.pone.0020649)
Supplement: Table S5 — χ2 tests for frequency distributions of alleles and genotypes in BIRC6 (Salt Lake City, Utah). (DOC) [file pone.0020649.s007.doc]

**Table S5: χ2 tests** for frequency distributions of alleles and genotypes in BIRC6 (Salt Lake City, Utah)

| **rsSNP and allele definitions** | **Samples** | **Allele 1**  **(freq)** | **Allele 2**  **(freq)** | **Fisher’s P-value (χ2)** | **OR (95% CI)** | **Genotype 11**  **(freq)** | **Genotype 12**  **(freq)** | **Genotype 22**  **(freq)** | **Fisher’s P-value (χ2)** | **HWE P-value Controls (χ2)** |
| --- | --- | --- | --- | --- | --- | --- | --- | --- | --- | --- |
| rs12612824 | POAG | 269(0.66) | 139(0.34) | 0.27 | 0.86 | 91(0.45) | 87(0.43) | 26(0.13) | 0.31 | 0.58 |
| 1=G 2=A | Control | 336(0.63) | 202(0.37) | (1.22) | (0.7-1.1) | 102(0.38) | 132(0.49) | 35(0.13) | (2.31) | (0.45) |
| rs17820747 | POAG | 309(0.76) | 97(0.24) | 0.22 | 0.82 | 116(0.57) | 77(0.38) | 10(0.05) | 0.40 | 0.22 |
| 1=A 2=C | Control | 426(0.80) | 110(0.20) | (1.5) | (0.6-1.1) | 166(0.62) | 94(0.35) | 8(0.03) | (1.84) | (1.51) |
| rs2069213 | POAG | 272(0.66) | 142(0.34) | 0.19 | 1.19 | 94(0.45) | 84(0.41) | 29(0.14) | 0.05 | 0.13 |
| 1=A 2=G | Control | 331(0.62) | 207(0.39) | (1.78) | (0.9-1.6) | 96(0.36) | 139(0.51) | 34(0.13) | (6.00) | (2.25) |
| rs2254106 | POAG | 229(0.55) | 185(0.45) | 0.45 | 1.11 | 67(0.32) | 95(0.46) | 45(0.22) | 0.68 | 0.64 |
| 1=G 2=A | Control | 312(0.58) | 228(0.42) | (0.58) | (0.8-1.4) | 92(0.34) | 128(0.47) | 50(0.19) | (0.77) | (0.22) |
| rs2754511 | POAG | 277(0.67) | 137(0.33) | 0.58 | 1.10 | 70(0.34) | 137(0.70) | 0(0.00) | 2.1E-06 | 0.07 |
| 1=A 2=T | Control | 352(0.65) | 188(0.35) | (0.31) | (0.8-1.4) | 108(0.40) | 136(0.54) | 26(0.10) | (26.25) | (3.25) |
