# Supplementary material for: Genes of the Unfolded Protein Response Pathway Harbor Risk Alleles for Primary Open Angle Glaucoma
Source: PLoS One. 2011 May 31;6(5):e20649. doi: 10.1371/journal.pone.0020649 (PMC3105107; doi:10.1371/journal.pone.0020649)
Supplement: Table S6 — χ2 tests for frequency distributions of alleles and genotypes in BIRC6 (San Diego, California). (DOC) [file pone.0020649.s008.doc]

**Table S6: χ2 tests** for frequency distributions of alleles and genotypes in BIRC6 (San Diego, California)

| **rsSNP and allele definitions** | **Samples** | **Allele 1**  **(freq)** | **Allele 2**  **(freq)** | **Fisher’s P-value (χ2)** | **OR (95% CI)** | **Genotype 11**  **(freq)** | **Genotype 12**  **(freq)** | **Genotype 22**  **(freq)** | **Fisher’s P-value (χ2)** | **HWE P-value Controls (χ2)** |
| --- | --- | --- | --- | --- | --- | --- | --- | --- | --- | --- |
| rs12612824 | POAG | 611 (0.65) | 329 (0.65) | 0.63 | 1.07 | 208 (0.44) | 195 (0.42) | 67 (0.14) | 0.39 | 0.65 |
| 1=G 2=A | Control | 193 (0.67) | 97 (0.33) | (0.23) | (0.8-1.4) | 63 (0.43) | 67 (0.46) | 15 (0.10) | (1.90) | (0.21) |
| rs17820747 | POAG | 703 (0.75) | 239 (0.25) | 0.97 | 1.01 | 261 (0.56) | 181 (0.38) | 29 (0.06) | 0.62 | 0.35 |
| 1=A 2=C | Control | 225 (0.75) | 77 (0.26) | (0.002) | (0.8-1.4) | 86 (0.57) | 53 (0.35) | 12 (0.08) | (0.94) | (0.87) |
| rs2069213 | POAG | 593(0.63) | 347(0.37) | 0.28 | 0.86 | 194(0.41) | 205(0.44) | 71(0.15) | 0.27 | 0.49 |
| 1=A 2=G | Control | 201(0.67) | 101(0.34) | (1.2) | (0.7-1.1) | 65(0.43) | 71(0.47) | 15(0.10) | (2.60) | (0.48) |
| rs2254106 | POAG | 564(0.60) | 378(0.40) | 0.09 | 0.80 | 178(0.38) | 208(0.44) | 85(0.18) | 0.23 | 0.63 |
| 1=G 2=A | Control | 164(0.54) | 138(0.46) | (2.94) | (0.6-1.0) | 46(0.30) | 72(0.48) | 33(0.22) | (2.90) | (0.23) |
| rs2754511 | POAG | 685(0.73) | 257(0.27) | 0.0006 | 1.62 | 257(0.54) | 171(0.36) | 43(0.09) | 0.0008 | 0.30 |
| 1=A 2=T | Control | 183(0.62) | 111(0.38) | (11.80) | (1.2-2.1) | 54(0.365) | 75(0.51) | 18(0.12) | (14.30) | (1.10) |
