# Supplementary material for: Genes of the Unfolded Protein Response Pathway Harbor Risk Alleles for Primary Open Angle Glaucoma
Source: PLoS One. 2011 May 31;6(5):e20649. doi: 10.1371/journal.pone.0020649 (PMC3105107; doi:10.1371/journal.pone.0020649)
Supplement: Table S7 — Estimated PDIA5 haplotype frequencies and association significance for the Salt Lake City population. (DOC) [file pone.0020649.s009.doc]

**TABLE S7**. Estimated PDIA5 haplotype frequencies and association significance for the Salt Lake City, Utah Population

| **Haplotypes** | **Case (Freq)** | **Control (Freq)** | **χ2** | **Fisher's P-value** | **Odds ratio (95% CI)** |
| --- | --- | --- | --- | --- | --- |
| GAAAAGAG | 6 (0.019) | 18 (0.037) | 2.0 | 0.15 | 0.51 (0.20-1.3) |
| GAAAAGGG | 0 (0.000) | 17 (0.041) | 11.1 | 0.001 | - |
| GAGAAGAG | 3 (0.010) | 20 (0.04) | 6.6 | 0.01 | 0.23 (0.07-0.80) |
| GAGAAGGG | 0 (0.000) | 40 (0.082) | 27.0 | 1.3E-07 | - |
| GAGAGGGG | 0 (0.000) | 17 (0.030) | 11.1 | 0.0009 | - |
| GTAAAAGG | 0 (0.000) | 19 (0.040) | 12.5 | 0.0004 | - |
| GTAAAGAG | 20 (0.059) | 32 (0.061) | 0.002 | 0.96 | 0.99 (0.55-1.77) |
| GTAAAGGA | 42 (0.132) | 37 (0.076) | 7.9 | 0.004 | 1.96 (1.2-3.1) |
| GTAAAGGG | 80 (0.252) | 68 (0.130) | 20.1 | 7.7E-006 | 2.4 (1.6-3.5) |
| GTAAGGGG | 20 (0.063) | 27 (0.055) | 0.28 | 0.60 | 1.1 (0.65-2.2) |
| GTAGAGGG | 25 (0.071) | 21 (0.046) | 4.8 | 0.03 | 1.9 (1.1-3.5) |
| GTGAAGAG | 11 (0.034) | 8 (0.016) | 3.4 | 0.06 | 2.3 (0.93-5.9) |
| GTGAAGGG | 8 (0.03) | 15 (0.03) | 0.18 | 0.67 | 0.83 (0.35-1.97) |

Haplotype frequencies <0.03 were excluded from the analysis
