# Supplementary material for: Genes of the Unfolded Protein Response Pathway Harbor Risk Alleles for Primary Open Angle Glaucoma
Source: PLoS One. 2011 May 31;6(5):e20649. doi: 10.1371/journal.pone.0020649 (PMC3105107; doi:10.1371/journal.pone.0020649)
Supplement: Table S8 — Estimated PDIA5 haplotype frequencies and association significance for the San Diego population. (DOC) [file pone.0020649.s010.doc]

**TABLE S8. Estimated PDIA5 haplotype frequencies and association significance for the San Diego, California Population**

| **Haplotypes** | **Case (Freq)** | **Control (Freq)** | **χ2** | **Fisher's P-value** | **Odds ratio (95% CI)** |
| --- | --- | --- | --- | --- | --- |
| GAAAAGAG | 64 (0.07) | 27 (0.10) | 2.3 | 0.13 | 0.69 (0.43-1.12) |
| GAAAAGGA | 48 (0.05) | 4.5(0.02) | 7.0 | 0.008 | 3.45 (1.3-9.3) |
| GAAAAGGG | 29 (0.03) | 20 (0.07) | 9.2 | 0.002 | 0.41 (0.23-0.73) |
| GAGAAGAG | 69 (0.07) | 6 (0.02) | 11.5 | 0.0007 | 4.0 (1.70-9.60) |
| GAGAAGGA | 23 (0.03) | 18 (0.07) | 10.80 | 0.001 | 0.36 (0.19-0.68) |
| GAGAAGGG | 90 (0.10) | 48 (0.17) | 10.3 | 0.001 | 0.52 (0.35-0.76) |
| GAGAGGAG | 22 (0.02) | 9 (0.03) | 0.35 | 0.55 | 0.79 (0.35-1.75) |
| GAGGAGGG | 38 (0.04) | 12 (0.04) | 0.002 | 0.96 | 0.99 (0.50-1.94) |
| GTAAAGGA | 24 (0.03) | 17 (0.06) | 8.6 | 0.003 | 0.4 (0.20-0.75) |
| GTAAAGGG | 82 (0.09) | 5 (0.02) | 18 | 2.2E-005 | 6.0 (2.4-16.3) |
| GTAGAGGG | 39 (0.04) | 13 (0.05) | 0.17 | 0.68 | 0.87 (0.46-1.64) |
| GTGAAGAG | 10 (0.01) | 12 (0.04) | 13.4 | 0.0002 | 0.23 (0.10-0.50) |
| GTGAAGGA | 31 (0.03) | 3 (0.01) | 4.9 | 0.03 | 3.8 (1.10-13.8) |
| GTGAAGGG | 49 (0.05) | 3 (0.01) | 10.0 | 0.002 | 5.7 (1.7-19.3) |
| GTGAGGGG | 31 (0.03) | 2 (0.01) | 4.8 | 0.03 | 3.9 (1.05-14.52) |

Haplotype frequencies <0.03 were excluded from the analysis
