# Supplementary material for: Genes of the Unfolded Protein Response Pathway Harbor Risk Alleles for Primary Open Angle Glaucoma
Source: PLoS One. 2011 May 31;6(5):e20649. doi: 10.1371/journal.pone.0020649 (PMC3105107; doi:10.1371/journal.pone.0020649)
Supplement: Table S9 — Estimated BIRC6 haplotype frequencies and association significance for the Salt Lake City population. (DOC) [file pone.0020649.s011.doc]

**TABLE S9. Estimated BIRC6 haplotype frequencies and association significance in the Salt Lake City, Utah population**

| **Haplotypes** | **Case (Freq)** | **Control (Freq)** | **χ2** | **Fisher's P-value** | **Odds ratio (95% CI)** |
| --- | --- | --- | --- | --- | --- |
| AAGGA | 108 (0.27) | 182(0.34) | 6.6 | 0.01 | 0.68 (0.51-0.91) |
| AAGGT | 20 (0.05) | 4(0.01) | 14.8 | 1.1E-04 | 6.1 (2.2-17.4) |
| GAAAA | 62 (0.16) | 53(0.10) | 6.2 | 0.01 | 1.64 (1.1-2.4) |
| GAAAT | 100 (0.25) | 157(0.30) | 2.9 | 0.09 | 0.77 (0.57-1.0) |
| GCAGA | 74 (0.19) | 81(0.15) | 1.7 | 0.19 | 1.26 (0.89-1.8) |

Haplotype frequencies <0.03 were excluded from the analysis
