# Supplementary material for: Genes of the Unfolded Protein Response Pathway Harbor Risk Alleles for Primary Open Angle Glaucoma
Source: PLoS One. 2011 May 31;6(5):e20649. doi: 10.1371/journal.pone.0020649 (PMC3105107; doi:10.1371/journal.pone.0020649)
Supplement: Table S10 — Estimated BIRC6 haplotype frequencies and association significance for the San Diego population. (DOC) [file pone.0020649.s012.doc]

**TABLE S10**. Estimated BIRC6 haplotype frequencies and association significance in the San Diego, California population

| **Haplotypes** | **Case (Freq)** | **Control (Freq)** | **χ2** | **Fisher's P-value** | **Odds ratio (95% CI)** |
| --- | --- | --- | --- | --- | --- |
| AAGGA | 312 (0.33) | 85 (0.30) | 0.98 | 0.32 | 1.1 (0.86-1.6) |
| GAAAA | 117 (0.13) | 32 (0.12) | 0.11 | 0.74 | 1.1 (0.70-1.60) |
| GAAAT | 236 (0.25) | 90 (0.32) | 5.70 | 0.02 | 0.7 (0.50-0.90) |
| GCAGA | 190 (0.20) | 47 (0.17) | 1.48 | 0.22 | 1.2 (0.90-1.70) |

Haplotype frequencies <0.03 were excluded from the analysis
